# The Evolving Concept of the Blood Brain Barrier (BBB): From a Single Static Barrier to a Heterogeneous and Dynamic Relay Center

**DOI:** 10.3389/fncel.2019.00405

**Published:** 2019-09-20

**Authors:** Andres Villabona-Rueda, Clara Erice, Carlos A. Pardo, Monique F. Stins

**Affiliations:** ^1^W. Harry Feinstone Department of Molecular Microbiology and Immunology, Malaria Research Institute, Johns Hopkins Bloomberg School of Public Health, Baltimore, MD, United States; ^2^Department of Neurology, Division of Neuroimmunology and Neuroinfectious Disorders, Johns Hopkins University School of Medicine, Baltimore, MD, United States

**Keywords:** cerebral endothelial cells, blood–brain barrier, neurovascular unit, brain cellular heterogeneity, vascular heterogeneity

## Abstract

The blood–brain barrier (BBB) helps maintain a tightly regulated microenvironment for optimal central nervous system (CNS) homeostasis and facilitates communications with the peripheral circulation. The brain endothelial cells, lining the brain’s vasculature, maintain close interactions with surrounding brain cells, e.g., astrocytes, pericytes and perivascular macrophages. This function facilitates critical intercellular crosstalk, giving rise to the concept of the neurovascular unit (NVU). The steady and appropriate communication between all components of the NVU is essential for normal CNS homeostasis and function, and dysregulation of one of its constituents can result in disease. Among the different brain regions, and along the vascular tree, the cellular composition of the NVU varies. Therefore, differential cues from the immediate vascular environment can affect BBB phenotype. To support the fluctuating metabolic and functional needs of the underlying neuropil, a specialized vascular heterogeneity is required. This is achieved by variances in barrier function, expression of transporters, receptors, and adhesion molecules. This mini-review will take you on a journey through evolving concepts surrounding the BBB, the NVU and beyond. Exploring classical experiments leading to new approaches will allow us to understand that the BBB is not merely a static separation between the brain and periphery but a closely regulated and interactive entity. We will discuss shifting paradigms, and ultimately aim to address the importance of BBB endothelial heterogeneity with regard to the function of the BBB within the NVU, and touch on its implications for different neuropathologies.

## Graphical Abstract

Heterogeneity of the cells of the neurovascular unit contributes to a significant vascular heterogeneity of the blood brain barrier endothelium in different brain regions, such as white matter and gray matter.

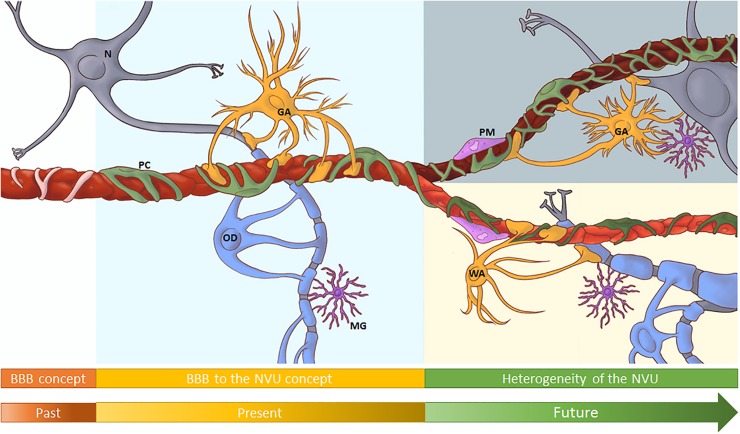

## The Blood Brain Barrier: Historical Perspective

The central nervous system (CNS) needs a highly controlled microenvironment for optimal functioning. Several barriers of the CNS, including the cerebral endothelial cells (CECs) of the blood brain barrier (BBB) tightly regulate transport into and out of the CNS. An early indication of a barrier at the cerebral blood vessels was recorded by Ridley (1653–1708) after injecting wax and mercury, resulting in three-dimensional vessel casts. While trying to improve histological tissue staining, the classical experiments by Ehrlich and Goldman, during the late 1800s and early 1900s, also suggested a separation between the CNS and peripheral circulation. During that same era, Lewandowsky tested neuro-pharmacologically active substances in animals, and observed neurological effects of only a subset. He hypothesized that, in order to shuttle them into the CNS, the brain vessel wall displayed a specific affinity for these select substances. Studies on the movement of substances between peripheral blood, cerebrospinal fluid and brain, led Lina Stern and collaborators (1918–1934) to the conclusion that CECs played a dual role in both protecting and metabolically supporting the CNS, thereby effectively proposing the BBB concept. The introduction of the transmission electron microscope allowed Reese and Karnovsky (1967) to show that electron dense tracers were not able to penetrate in-between adjacent CECs, hence pointing to the actual site of the barrier. Subsequent studies with tracers and micro-electrodes, confirmed the low BBB permeability, and demonstrated its high *trans-*endothelial electrical resistance (Crone and Olesen, 1982). In the late 1980s it was discovered that transmembrane multi-protein tight junctional complexes at CEC-CEC borders conferred to the BBB its barrier function. Freeze facture studies, initially carried out by Farquhar and Palade (1963), revealed the complex belt-like networks of these cell–cell junctions. Analysis, first on epithelial cells and later confirmed for brain endothelium, identified individual junctional components, including claudins, occludins, junctional adhesion molecules, AJ (e.g., VE-cadherin, N-cadherin, and β-catenin) and cytoplasmic adaptors, such as zona occludens proteins. Due to its stringent barrier function and low vesicle transport activity, the passage of nutrients and waste products across the BBB was found to be regulated by polarized transporters on CECs; with efflux transporters, such as the ATP-binding cassette transporter family, usually at the luminal membrane, and solute carriers delivering essential nutrients into the CNS, such as GLUT-1, predominantly localized on the abluminal side (Bendayan et al., 2006; Roberts et al., 2008). Together, these classical experiments established the concept of a tight BBB at endothelial junctions. In-depth reviews on the history of the BBB (Liddelow, 2011; Saunders et al., 2014) and BBB-endothelial junctions (Haseloff et al., 2015; Bauer and Traweger, 2016; Stamatovic et al., 2016) are suggested.

**FIGURE 1:**
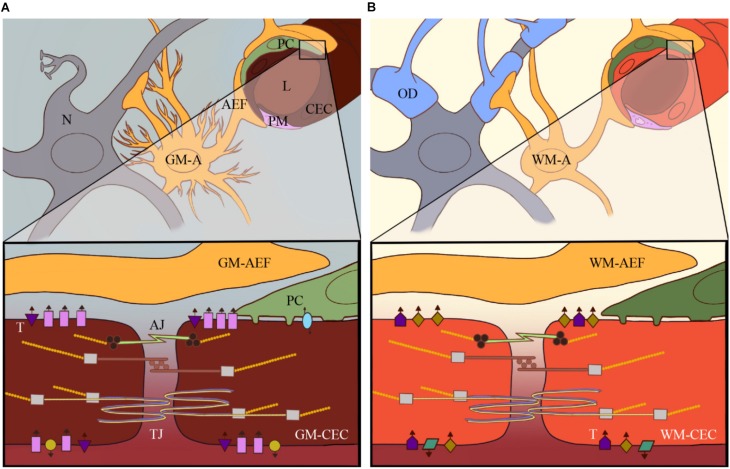
Heterogeneity of the neurovascular unit (NVU): current research has revealed high heterogeneity of e.g., astrocytes and pericytes within the CNS and among different brain areas. The heterogeneity of these different cellular components of the NVU in, for example, the GM **(A)** versus the WM **(B)** contributes to brain vascular heterogeneity to support local physiological and metabolic needs for that particular part of the brain. This includes differential expression of specific receptors and transporters on CECs, such as GLUT-1, Pgp and Na^+^/K^+^-ATPase, which are represented by the different shapes and colors in GM **(A)** versus WM-CECs **(B)**.

## Established Principles: From the BBB to the Neurovascular Unit

### Influence of Brain Cells on CECs

The modulating influence of the neural environment on the BBB was first suggested by Stewart and Wiley (1981) in a series of reverse grafting experiments of neural and non-neural tissues. Ultrastructural studies revealed close apposition of AEF to CECs, and cell transplantation experiments and trypan blue exclusion confirmed that astrocytes contributed to the BBB phenotype (Janzer and Raff, 1987). Subsequent studies showed that astrocyte-derived signaling to CECs leading to increased expression of tight-junctions was mediated by, e.g., transforming growth factor beta (TGFβ), Sonic hedgehog and Wnt signaling (Siddharthan et al., 2007; Alvarez et al., 2011; Wang et al., 2018; Benz et al., 2019). Moreover, astrocytes regulate expression of alkaline phosphatase and Na-ATPase on CECs via cAMP and IL6, suggesting astrocytes regulate ionic homeostasis (Beuckmann et al., 1995; Sun et al., 1997).

Besides astrocytes, pericytes also influence the BBB phenotype. Pericytes are mural cells that wrap around the abluminal surface of cerebral microvessels. Due to the presence of smooth muscle-like fibers, contractile characteristics and expression of vaso-active mediators, pericytes were initially thought to regulate microvascular hemodynamics (Balabanov and Dore-Duffy, 1998). Subsequent ultrastructural studies revealed that pericytes intercalate with AEF, covering up to 60–70% of CECs, and maintain close physical contacts with CECs via gap junctions and “peg-socket” structures, indicative of communicative functions. In the CNS, pericyte loss and low pericyte coverage correlate with increased BBB permeability, implicating their involvement in regulating BBB-barrier functions (Winkler et al., 2011). Combined *in vivo* murine models and *in vitro* studies confirmed the ability of pericytes to directly modulate BBB phenotype by regulating, e.g., Wnt and Notch signaling, caveolar transport across the BBB through the expression of the lysolipid transporter mfsd2a (Ben-Zvi et al., 2014; Sweeney et al., 2016), and the expression of GLUT-1 and transferrin receptor CD71 (Liebner et al., 2011). Pericyte CD146, together with PDGF act via TGFβ, contributing to CECs barrier function (Armulik et al., 2010; Sa-Pereira et al., 2012; Chen et al., 2017). Indirectly, pericytes target CECs by inducing polarization of AQP4, Kir4.1, and laminin-α2 on AEF and thus indirectly affect permeability by restricting vesicular transport across CECs (Hori et al., 2004; Armulik et al., 2010; Daneman et al., 2010). *In vitro* data suggests pericyte involvement in immune function, through modulation of phagocytosis, expression of αSMA and ICAM-1 and supporting ICAM-1-mediated neutrophil transmigration in response to pro-inflammatory stimuli and the generation of mediators such as iNOS, ROS, COX2, MHCII, (Pieper et al., 2014). Reviews on pericyte-neurovascular unit (NVU) interactions (De Bock et al., 2017; Zhao et al., 2018) and signaling (Sweeney et al., 2016) are suggested for more in-depth information.

Neuronal effects on the BBB-CEC phenotype include release of growth factors, such as neuregulin and brain-derived neurotrophic factor, (Gauthier et al., 2013). Neuronal activity and neurotransmitter release can regulate BBB permeability through, e.g., glutamate-activating CEC-NMDA receptors and modulate transport of insulin-like growth factor, across the BBB (Nishijima et al., 2010; Vazana et al., 2016).

Taken together, the recognition of the contribution of inter-cellular communication between astrocytes, pericytes and neurons to the specific phenotype of CECs led to the formulation of the concept of the NVU. This model emphasizes the maintenance of CNS homeostasis through multidimensional, continuous and reciprocal communication among all NVU members by means of either physical contacts and/or the release of signaling mediators. Hence, dysregulation of one of the NVU components could lead to neuro-disease (Iadecola, 2017; Sweeney et al., 2019).

## Shifting Paradigms: From a Single NVU to a Heterogeneous NVU

### Heterogeneity of NVU Components

Differences in morphology, cellular content, and microvascular density have been observed among different brain regions and are especially apparent in white matter (WM) versus grey matter (GM). Historically, astroglial classifications were based on their morphology and anatomical position; as is the case with “fibrous astrocytes” with long processes in WM and more star-shaped “protoplasmic astrocytes” in GM. However, GM and WM astrocytes have extensive functional differences, as indicated by differential expression of transporters, including glutamate-transporters and GLUT-1 subtypes. In addition, they respond differently to *in vitro* stimuli (de Graaf et al., 2001; Goursaud et al., 2009). Hippocampal astroglia display differential ion channel expression and GABA responses (Cavaglia et al., 2001), confirming the regional heterogeneity of astroglial populations. The overall transcriptomes of GM and WM were shown to be unique and corroborate functional heterogeneities (Mills et al., 2013). Further development of technical abilities and “big data” processing revealed a large heterogeneity of individual NVU members. RNA-seq studies of populations of brain cells exposed high heterogeneity in both morphology of astrocytes, glia, endothelial cells and pericytes and in physiological properties, metabolic processes and functions (Zhang and Barres, 2010; Zhang et al., 2014; Li et al., 2019).

Single cell transcriptomics also indicated high diversity of glial sub-populations throughout the CNS, including in immunologic profiles (Batiuk et al., 2018). Clear region-specific differences, with a predominance of type-I interferon genes in GM, versus NFκB-signaling in WM was observed. Similarly, RNAseq showed that, with respect to immunological responses, isolated microglia clustered into at least nine transcriptionally different states (Hammond et al., 2019). Likewise, transcriptional profiling highlighted large differences in microglial populations derived from WM versus GM, with amoeboid-type microglia in WM regions (Verdonk et al., 2016; van der Poel et al., 2019). Transcriptional profiling also provided additional insights into the heterogeneity of the spatiotemporal responses of microglia to disease (Masuda et al., 2019). For example, metabolically active amoeboid microglia with phagocytic capability share gene signatures with those associated with degenerative disease (Li et al., 2019; Staszewski and Hagemeyer, 2019). These diverse populations also have different roles during neuronal plasticity as shown by their variances in response to injury and disease (Tay et al., 2018; Masuda et al., 2019). Such diversity may differentially affect phenotype and function of CECs, thus contributing to vascular heterogeneity.

Perivascular macrophages are differentially distributed along the vascular tree. They aid in preserving BBB integrity and contribute to regulating vascular tone (Goldmann et al., 2016; Hoeffel and Ginhoux, 2018). Under inflammatory conditions, PMs respond quickly but differentially (He et al., 2016; Verdonk et al., 2016; van der Poel et al., 2019). However, their interactions within the NVU and their implications for BBB-phenotype are under-investigated. The pericyte population is heterogeneous, not just between macro- versus micro-vessels but also among capillaries. For example, pericyte arms are fewer and shorter in post-capillary venules, leading to differences in pre- versus post-capillary contraction capacity (Itoh and Suzuki, 2012). Pericyte coverage differs among brain regions affecting BBB permeability in a regional-dependent manner, although other mechanisms are involved (Winkler et al., 2018). Coverage is also higher in the cerebral cortex compared to the spinal cord, suggesting region-dependent differential regulation of the CECs phenotype and function (Wilhelm et al., 2016). As alluded above, pericyte-derived factors directly influence BBB function and affect AEF polarization, thus indirectly restricting vesicular transport across CECs. Although RNA-seq studies indicated heterogeneity among pericytes (Zhang et al., 2014), it is essential to exploit novel methods for isolation, characterization and analysis of pericytes from different brain regions. This will shed more light on the functional heterogeneity of pericytes, as well as on their contributions to vascular heterogeneity (Crouch and Doetsch, 2018; Dore-Duffy and Esen, 2018).

Oligodendrocytes are more prevalent in WM compared to GM brain regions, therefore their function was traditionally viewed as to myelinate neuronal processes and facilitate neural transmission. In the 1920s Rio Hortega described four types of oligodendroglia, based on the number of axons they myelinated and their location; perineural or perivascular. Recently, single-cell RNA sequencing with Fluidigm-C1 technology revealed 12 clusters of heterogeneous oligodendrocyte populations or states. Their functional heterogeneity among brain regions may be related to different progenitor lineages (Dimou and Simons, 2017; Trotter and Mittmann, 2019). Although little is known about the communication between oligodendrocytes and CECs, the survival and proliferation of oligodendrocyte precursor cells is influenced by factors released from CECs, such as brain-derived neurotropic growth factor (Arai and Lo, 2009; Hamanaka et al., 2018). Oligodendrocyte progenitors can modulate BBB integrity via secretion of TGFβ-1, resulting in the upregulation of junctional proteins in CECs (Seo et al., 2014). Pericytes also influence progenitor development and neuronal myelination via Lama2 and VEGF signaling and by regulating the bioavailability of PDGF and TGFβ (De La Fuente et al., 2017; Girolamo et al., 2019). Due to the high oligodendrocyte prevalence in WM, CECs in WM are likely to have differential cellular interactions than CECs residing in GM. More research on the interactions and communications between oligodendrocytes and CECs and the consequences for specific phenotypes of CECs in WM is needed.

### Physiological Heterogeneity

Besides cellular interactions within the NVU, physiological differences, such as blood flow, affect CEC-vascular phenotype. For the brain’s blood supply, large vessel branches penetrate the brain parenchyma, morphing into a dense network of small arteries, arterioles, capillaries, and venules. Compared to larger vessels, the microvasculature does not contain smooth muscles but pericytes, indicating differences in regulation of vaso-reactivity, blood flow, and shear stress (Cipolla, 2016; Mikhail Kellawan et al., 2017). Shear stress has been shown to affect expression of transporters, ion channels, and of tight- and adherens junction proteins on CECs (Cucullo et al., 2011).

Dependent on brain area, the vasculature displays differential densities and spatial orientation. In mouse frontal cortex, GM vessels are perpendicular to the pyramidal cell layer, whereas WM vessels are orientated parallel to axonal fibers (Itoh and Suzuki, 2012; Murugesan et al., 2012). The capillary density in the GM is greater than in the WM, reflecting different regional metabolic and energy demands, such as in synaptically active regions (e.g., cerebral cortex) versus fiber tract heavy regions (e.g., corpus callosum) (Cavaglia et al., 2001; Itoh and Suzuki, 2012; Wilhelm et al., 2016). To support metabolic needs in areas with low vascular density, an increased presence of transcellular pathways, gap-junctions and specific expression of receptors and transporters on CECs is needed (Cavaglia et al., 2001; Keaney and Campbell, 2015). Along the vascular tree, from large to small vessels, heterogeneity in the expression of various genes and expression of claudin-5 is evident (Macdonald et al., 2010; Paul et al., 2013; Sabbagh et al., 2018). Transcripts for junctional proteins occludin, claudin-5, and α-catenin were increased in WM-CECs compared to GM-CECs (Nyul-Toth et al., 2016).

The physiological/metabolic needs of the highly active neural milieu are in constant flux. Demands for exchange of nutrients, solutes, water and oxygen are conveyed through cues to the brain microvasculature. As discussed above, there is high diversity in the cellular composition of the NVU, which includes a significant heterogeneity of astrocytes, pericytes and oligodendrocytes in different brain areas. Taking into account the reciprocal interactions of these brain cells within the NVU and differing metabolic needs and differences in blood flow/shear stress, the microvascular phenotype *must* differ between different brain regions, especially between WM and GM regions. Characterizing and understanding the implications of regional heterogeneity of the brain microvasculature in health and disease is a new frontier for brain vascular research.

## Future Perspectives: Implication of Vascular Heterogeneity for Neuropathologies and *In Vitro* Modeling

The critical role of brain vascular pathology recently emerged in studies of various neurological diseases, e.g., multiple sclerosis, infections including cerebral malaria and HIV, neurodegenerative disorders, including Alzheimer’s disease, traumatic brain injuries and in some psychiatric diseases. Small vessel disease exhibits brain vascular pathologies associated with either focal or generalized changes in different brain regions (Varghese et al., 2017; Kealy et al., 2018), comprised of WM lesions, cerebral micro-bleeds (Pantoni, 2010) with BBB opening and vasogenic edema resulting from oxygen loss and vascular inflammatory responses, including MMP release (Yang and Rosenberg, 2011). In multiple sclerosis, microvascular centered inflammatory lesions involve BBB breakdown and are associated with foci of demyelination in both GM and WM regions (Prins et al., 2015). In WM, early demyelination, the loss of WM volume and leukocyte infiltrations are associated with local BBB damage (Lucchinetti et al., 2011; Popescu et al., 2011; Prins et al., 2015; Granberg et al., 2017). Cerebral malaria, a severe neurological complication of malaria, and post-CM sequelae present a clear example of differential WM and GM pathology associated with BBB permeability and hemorrhagic punctae which predominate in WM but not in GM (Taylor and Molyneux, 2015). Alzheimer’s is regarded mainly as a GM disease with heterogeneous findings that include small vessel damage, cerebral amyloid angiopathy, inflammation, and hypercoagulability (Zamolodchikov and Strickland, 2016). In addition, differences in vascular pathologies between different lobes and WM versus GM are reported, with the occipital lobe more severely affected by cerebral amyloid angiopathies, followed by frontal-, temporal-, and parietal lobes (Vinters et al., 1996; Attems and Jellinger, 2014). Recent studies show clear vascular pathologies in traumatic brain injury, involving neurovascular inflammation, ROS, MMP’s resulting in a loss of junctional integrity (Abdul-Muneer et al., 2015). Major depressive disorder and attention deficit-hyperactivity disorder also exhibit signs of vascular pathology. In schizophrenia, evidence is mounting for NVU involvement (Najjar et al., 2017). Here, selected regions of GM are predominately affected (Cannon et al., 2002; Vita et al., 2012), whereas WM involvement is limited to select tracts (Davis et al., 2003; Hercher et al., 2014). Postmortem brain samples of schizophrenic patients also revealed ultrastructural differences in brain capillaries (Uranova et al., 2010).

Several brain diseases have underlying vasculopathic mechanisms and the vascular heterogeneity may lead to differential neuropathologies, especially in GM versus WM. To understand the contribution of the brain’s vascular heterogeneity and dysregulation of the NVU to neuro-pathogenesis, besides animal models, appropriate *in vitro* models are needed. Traditionally, isolated single CECs cultures or combinations with astrocytes and/or pericytes have been used for *in vitro* BBB modeling. More recently, inducible progenitor cells have been used for BBB-models, which show a low permeability and express drug transporters. An additional advantage is that patient material can be differentiated into homologous multi-cell BBB-models (DeStefano et al., 2018). However, considering NVU-cell heterogeneity, it is not clear which part of the BBB vasculature is represented; e.g., WM or GM. Depending on the scientific questions involved, “simple” *in vitro* BBB models may suffice or to better recapitulate the complexity of the NVU interactions, multi-cellular, multi-compartment micro-fluidic models, or organ-on-a-chip approaches may be more suitable (Noumbissi et al., 2018). However, the incorporation of NVU heterogeneity, including diversity of astrocytes and pericytes derived from GM versus WM areas and the potential role of oligodendrocytes, has thus far, been neglected in BBB-model designs. When studying neuropathologies that differ in their presentation in various brain regions, it is particularly important to benchmark *in vitro* BBB models to the *in vivo* vasculature of the region of interest. Therefore, considering brain cell heterogeneity in experimental design may lead to BBB models better reflecting WM versus GM vasculature.

## Concluding Remarks

This mini-review aims to highlight the region-specific heterogeneity of the brain’s vasculature and is not meant to be an exhaustive list. Additional BBB topics, including other barrier sites, immune interactions or BBB-development have been recently reviewed (Forrester et al., 2018; Mastorakos and McGavern, 2019; Saunders et al., 2019). Understanding the contributions of cellular diversity of the NVU micro-environment to phenotypic and functional heterogeneity of the brain’s vasculature will aid in elucidating differing region-dependent neuropathologies. This can be achieved by combining basic research and clinical approaches with large scale genetic/RNA-seq and proteomic analysis of regional microvasculature and other NVU components. Ultimately, this may inform us of novel targets for designing region-specific neuro-therapeutics.

## Key Concepts

### Blood–Brain Barrier

Cerebral endothelial cells forming a low permeability barrier between the peripheral blood circulation and the CNS. Presence of TJ and polarized transporters tightly regulate passage of molecules into and out of the CNS.

### Neurovascular Unit

The concept that CECs, astroglial cells, pericytes, PMs, and neurons communicate together to maintain brain homeostasis for optimal functioning of the organism. Dysfunction of any one component affects another and can lead to neuro-disease.

### Brain Cellular Heterogeneity

Heterogeneity in morphological-, molecular phenotype, and function of brain cells (e.g., glia, neurons). As these cells interact with the brain vascular CECs, they can influence the CECs phenotype and function, reflecting the needs of the underlying brain tissue.

### Vascular Heterogeneity

Variances in the anatomical, cellular and molecular composition of the vasculature. Differential interactions with adjacent brain tissue can lead to a heterogeneity of the BBB phenotype and function not only along the vascular tree, e.g., large to small vessels, but also in different brain regions.

## Author Contributions

AV-R, CE, and MS wrote the manuscript. MS and CP conceptualized and edited the manuscript. All authors contributed equally to this manuscript.

## Conflict of Interest Statement

The authors declare that the research was conducted in the absence of any commercial or financial relationships that could be construed as a potential conflict of interest.

## Abbreviations

AEF: astrocyte endfeet
AJ: adherens junctions
GM-A: gray matter astrocyte
GM-CEC: cerebral endothelial cell in gray matter
L: lumen of brain microvessel
N: neurons
OD: oligodendrocyte
PC: pericyte
PM: perivascular macrophage
T: transporters
TJ: tight junctions
WM-A: white matter astrocyte
WM-CEC: cerebral endothelial cell in white matter
